# Integrated meta-analysis of human astrocytes transcriptomes reveals a candidate recurrent inflammatory signature in response to inflammatory and immune stimuli

**DOI:** 10.3389/fncel.2026.1870142

**Published:** 2026-07-29

**Authors:** Andrea Luque-Bolivar, Karem Ruiz-Araujo, Andrés Felipe Aristizábal-Pachón, Janneth González

**Affiliations:** 1 Departamento de Nutrición y Bioquímica, Facultad de Ciencias, Pontificia Universidad Javeriana, Bogotá, Colombia; 2 Foundation for Clinical and Molecular Applied Cancer Research (FICMAC), Bogotá, Colombia

**Keywords:** astrocytes, meta-analysis, neuroinflammation, RNA-seq, robust rank aggregation, transcriptome profiling

## Abstract

Astrocytes are key regulators of inflammatory and immune responses in the central nervous system, particularly under pathological conditions. We conducted a systematic search of the NCBI GEO and ENA databases to identify transcriptomic studies of stimulated astrocytes. This meta-analysis integrates 11 RNA-Seq datasets, encompassing a total of 153 samples (91 stimulated, and 62 controls) exposed to pro-inflammatory stimuli such as cytokines (TNF-α, IL-6, and IL-1β), palmitic acid, and pathogens like SARS-CoV-2 and *Borrelia burgdorferi*. Through robust rank aggregation (RRA), we identified 130 differentially expressed genes (DEGs), including 125 upregulated and 5 downregulated. Functional enrichment analyses revealed that these DEGs are primarily involved in immune and inflammatory pathways, such as cytokine signaling, interferon responses, and NF-κB activation. Network analysis revealed five hub nodes, CXCL10, DDX58, IFIH1, IL-1β, and TLR3, underscoring their importance in astrocytic inflammatory signaling. These findings emphasize the ability of astrocytes to act as immunocompetent cells that coordinate inflammatory responses through mechanisms such as the NOD-like receptor and NF-κB pathways. Although chronic activation of NF-κB has been linked to inflammation, this pathway also plays essential roles in synaptic plasticity. Moreover, the consistent upregulation of *DDX58* and *IFIH1* across varied inflammatory stimuli suggests that astrocytes transition into a common ‘reactive’ state that may contribute to chronic neuroinflammation. This study identifies a candidate gene signature and underscores the dual protective and pathological roles of astrocytes in inflammatory processes.

## Introduction

Astrocytes are glial cells that play essential roles in the normal functioning of the central nervous system (CNS). Their diverse functions in maintaining brain homeostasis include regulating blood flow, providing metabolic and antioxidant support to neurons, secreting pro- and anti-inflammatory molecules, regulating ionic and neurotransmitter balance, and controlling immune cell activation (Baxter et al., 2021; Liddelow and Barres, 2017; Mahmoud et al., 2019; Patani et al., 2023). In addition, as immunocompetent cells, astrocytes can express damage-associated molecular patterns (DAMP) and pathogen-associated molecular pattern (PAMP) receptors by undergoing “reactive transformation” (Gong et al., 2020).

Reactive astrogliosis represents a defensive process in which astrocytes undergo a series of changes to minimize and repair damage. During such reactive states, astrocytes experience morphological, molecular, cellular, and functional changes, reflecting the heterogeneity of their responses to various factors. For example, reactive astrocytes can exert neuroprotective effects by promoting blood–brain barrier (BBB) repair, sequestering infections via glial scarring, and facilitating metabolic support for surviving neurons (Lawrence et al., 2023) However, this reactivity can also generate detrimental effects if an imbalance occurs between their neuroprotective and neurotoxic effects, which is often related to the intensity and duration of the inflammatory response (Colombo and Farina, 2016; Matusova et al., 2023; Sofroniew, 2025).

Neuroinflammatory responses are driven primarily by a variety of mediators, including cytokines (e.g., IL-1β, IL-6, and TNF-α), second messengers, and reactive oxygen species. Although multiple cell types contribute to the production of these mediators, including microglia, peripheral macrophages, and neurons, astrocytes stand out for their ability to functionally integrate into these processes. Specifically, their strategic localization, functional plasticity, and capacity to amplify inflammatory signals enable them to actively participate in the initiation, modulation, and resolution of neuroinflammation, positioning them as central regulators of the neuroimmune environment (Ben Haim et al., 2015). In addition to signaling, these mediators are essential for neuronal metabolism, immune survival signaling, leukocyte trafficking and other neuroinflammatory processes. Consequently, their dysregulation is a critical factor in the progression of neuroinflammation, neurodegeneration, and demyelination within the CNS (Cieri and Ramos, 2025; Naveed et al., 2025). For example, during chronic or pathological neuroinflammation induced by infection, injury, or insult, the integrity of the BBB is compromised by immune cell infiltration, edema, cell death, and increased permeability. As neuroinflammation is associated with severe pathological conditions and may trigger autoimmune responses or neurodegenerative disorders, there is a crucial need to investigate the reactivity and molecular mechanisms underlying the response of astrocytes to different stimuli (Hawkins and Davis, 2005; Michael et al., 2026; Monahan et al., 2008)

Reactive astrocyte states are typically characterized by phenotypic changes, such as hypertrophy and increased immune reactivity of glial fibrillary acidic protein (GFAP) (Cieri et al., 2023). While initial frameworks in murine models proposed a dichotomous classification of astrocyte reactivity, distinguishing neurotoxic (A1) and neuroprotective (A2) states, emerging transcriptomic evidence reveals a highly diverse spectrum of activation states. This molecular diversity is tailored to the nature of the stimulus, regional heterogeneity and species-specific genomic architectures (Cunningham et al., 2019; González-Giraldo et al., 2021; Liddelow and Barres, 2017). Consequently, current international consensus guidelines underscore that the binary A1/A2 nomenclature oversimplifies the multidimensional nature of reactivity (Escartin et al., 2021)

Despite the abundance of stimulus-specific transcriptomic data, a definitive, cross-stimulus ‘core’ signature of human astrocyte reactivity remains elusive. By integrating diverse RNA-seq datasets, this meta-analysis aims to uncover a candidate recurrent inflammatory gene signature that may be shared across reactive astrocyte states, providing a basis for future investigations. To this end, we employed Robust Rank Aggregation (RRA) to synthesize transcriptomic responses derived from 11 independent human astrocyte datasets. This approach aims to establish a robust gene signature of neuroinflammation that effectively minimizes the influence of individual study bias and stimulus-specific variability, ensuring the biological reproducibility of the transcriptional features.

## Methodology

### Research strategy and inclusion and exclusion criteria

**FIGURE 1:**
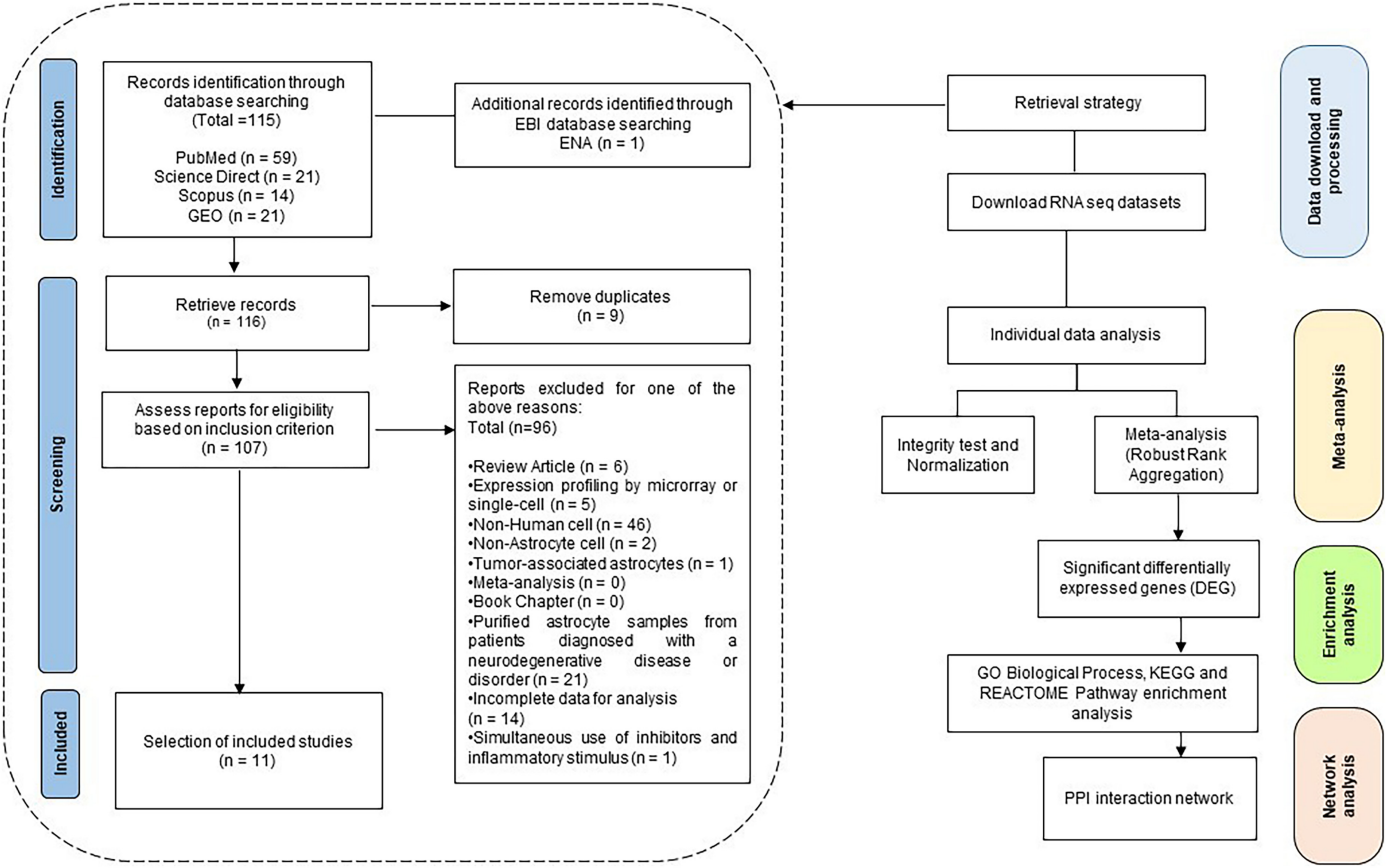
Study selection and transcriptomic meta-analysis workflow. Flow diagram illustrating the systematic screening and selection of public transcriptomic datasets. Overview of the analytical pipeline used for the transcriptomic meta-analysis. The process includes individual data analysis, meta-analysis using RRA, and subsequent enrichment and network analysis. DEG: differentially expressed gene, EBI: European Bioinformatics Institute, GEO: gene expression omnibus, GO: gene ontology, KEGG: Kyoto encyclopedia of genes and genomes, PPI: protein–protein interaction, RRA: robust rank aggregation.

The study selection process was conducted according to the PRISMA 2020 guidelines (Page et al., 2021), as detailed in Figure 1. A comprehensive systematic search was conducted across multiple databases, including PubMed, Scopus, and ScienceDirect, as well as genomic repositories such as the Gene Expression Omnibus (GEO) and the European Nucleotide Archive (ENA). Specific Boolean strings were tailored for each database (e.g., PubMed: ((Astrocytes [MeSH Terms] OR ‘human astrocytes’[Title/Abstract]) AND (‘Sequence Analysis, RNA’[Mesh] OR ‘RNA-seq’[Title/Abstract] OR ‘transcriptome’[Title/Abstract] OR ‘RNAseq’[Title/Abstract])) AND (Interleukins[MeSH Terms] OR ‘cytokine’[Title/Abstract] OR ‘neuroinflammation’[Title/Abstract] OR ‘reactive astrogliosis’[Title/Abstract]) NOT (‘Neoplasms’[Mesh] OR ‘Astrocytoma’[Mesh]). The complete search strategy, including specific terms per database, is provided in Supplementary Table 1.

To be included in the study, the following criteria were used: (1) original article published between 2017 and 2023; (2) experimental design with paired samples, where each stimulated sample was matched to its corresponding control from the same biological source; (3) model of human astrocytes derived from pluripotent stem cells, purified fetal, or commercial cell lines; (4) stimulation with molecules or agents that promote the inflammatory process; (5) transcriptomic profiling via RNA-seq; and (6) availability of raw RNA-seq data in the GEO or EBI (European Bioinformatics Institute) databases. Studies were excluded if they met any of the following criteria: (1) book chapters and reviews; (2) experimental design without paired samples; (3) tumor or other nervous tissue cell models; (4) samples from astrocytes purified from patients diagnosed with a neurodegenerative disease or disorder; (5) studies in which astrocytes had been genetically manipulated prior to the inflammatory stimulus; and (6) transcriptomic profiles obtained from microarrays or single-cell, as illustrated in Figure 1.

For screening and selection studies, the titles and abstracts were independently reviewed by two reviewers (AL-B & KR) to identify potentially relevant studies that met the previously described eligibility criteria. Any disagreements between reviewers were resolved through consensus or with the help of a third evaluator (AF-AP).

### RNA-seq data extraction and preprocessing

After articles were selected based on the search strategy and criteria, raw sequencing read files (.fastq) were downloaded from the GEO and EBI databases for each study. All bioinformatics analyses were performed using the Galaxy platform (version 25.1.1. dev0) (Galaxy Community, 2024). The complete computational workflow, including tool versions and parameters, is available as a Galaxy workflow file (.ga format) in the GitHub repository^1^. An initial quality analysis of the reads was conducted to determine the presence of adapters and overrepresented reads using the FASTQC tool v0.12.1 (Andrews, 2010). Adapter trimming and low-quality reads were filtered out via Trimmomatic v0.39 (Bolger et al., 2014). Subsequently, ribosomal RNAs (rRNAs) were removed with the SortMeRNA tool v4.3.6 using the rRNA, Rfam and SILVA databases (Supplementary Table 2) (Kopylova et al., 2012). Nonribosomal RNAs were aligned to the reference genome obtained from GENCODE (GRCh38 version 44) with the STAR tool v2.7.10b (Dobin et al., 2013). Gene-level counts from reads mapped to the genome were quantified via the HTSeq-count package (Anders et al., 2015). Differential expression analysis was performed with the Limma-Voom package (Law et al., 2014). The trimmed mean of M values (TMM) normalization method was employed to correct for differences in overall expression. For each dataset, a design matrix based on the experimental condition was constructed, and treated samples were compared against their corresponding controls. The Benjamini–Hochberg method was employed to calculate adjusted *p*-values. This workflow was performed to minimize the degree of technical heterogeneity between studies.

### Robust rank aggregation (RRA)

For the Robust Rank Aggregation (RRA) analysis, genes were ranked according to log fold change (logFC) values obtained using the Limma-Voom package. Datasets containing multiple treatment conditions contributed one ranked gene list for each treatment-versus-control comparison. The ranked gene lists were then aggregated using the “RobustRankAggreg” package (version 1.2.1) (Kolde et al., 2012). This method employs an adjusted p-value to assess the likelihood that differentially expressed genes (DEGs) are consistently identified across datasets with highly ranked genes. Genes with an adjusted *p*-value < 0.05 and | log2FC| > 1 were considered significant DEGs in the RRA analysis. This method was selected based on the criteria that conditions of the experimental analysis varied. Differentially expressed genes were visualized using Morpheus tool^2^, which were stratified according to astrocyte source, including human induced pluripotent stem cell-derived astrocytes, fetal and primary astrocytes.

### Functional enrichment and pathway analysis

Gene Ontology (GO) and pathway enrichment analysis were performed for the identified DEGs from the RRA analysis (as defined by adjusted *p*-value < 0.05 and | log2FC| > 1, see RRA section), using gProfiler tool^3^ (Kolberg et al., 2023). The enrichment calculations were performed using the default background implemented by g:Profiler for the selected organism. g:Profiler analysis was performed including GO biological process, Reactome and KEGG pathways. Statistical significance was assessed using the g:SCS multiple testing correction method, with a significance threshold of adjusted *p* < 0.05. Additionally, the REVIGO tool^4^ was used to remove redundant GO terms, emphasize representative terms that group semantically similar terms, and prioritize statistically significant terms (Supek et al., 2011). From this clustering, biological processes related to immune and inflammatory responses were selected to identify the genes directly involved in these responses.

### Protein–protein interaction network

A protein–protein interaction (PPI) network was constructed via STRING (v.12.0) (Szklarczyk et al., 2023) as a downstream exploratory analysis to prioritize highly connected genes within the candidate inflammatory and immune-associated signature. All possible interaction sources that STRING database offers were included; these were “Text mining”, “Experiments”, “Databases”, “Co-expression”, “Neighborhood”, “Gene Fusion” and “Co-occurrence”. A minimum STRING confidence score of 0.4 was used, retaining interactions with confidence scores ≥ 0.4. The PPIs reported in STRING were visualized via Cytoscape (v.3.10.2) (Shannon et al., 2003) software. Key centrality measures including degree, betweenness centrality, eccentricity, average shortest path length, clustering coefficient and topological coefficient, were calculated. Network topology analysis was performed to identify candidate central nodes using betweenness centrality and degree distribution metrics. The degree method was employed to select nodes with the highest connectivity and betweenness centrality to quantify the number of times that a node lies on the shortest path connecting two other nodes; nodes with a high betweenness are considered more biologically informative that are responsible for transferring communication information (Opsahl et al., 2010).

Additionally, to identify functional modules within the network, the Molecular Complex Detection (MCODE) plug-in (v.2.0.3) (Bader and Hogue, 2003) was applied. This approach enabled to find functional subunits that may represent biological mechanisms. The parameters used for MCODE analysis, as default, were: degree cutoff = 2, node score cutoff = 0.2, k-core = 2 and max depth = 100. An enrichment analysis was performed using the STRING database. A 0.05 False Discovery Rate (FDR) threshold was applied for statistical significance and only terms with a minimum signal strength of 0.01 were included. Terms were considered only if at least two genes were present in the network. Enriched terms were grouped based on a similarity threshold of ≥0.8.

## Results

Following the search strategy, 11 RNA-Seq datasets were selected and are detailed in Table 1. In total, 153 samples were analysed, comprising 91 stimulated samples and 62 controls. The stimuli used included proinflammatory molecules (TNF-α, IL-6, IL-1β, palmitic acid, and FBS) as well as pathogens such as viruses and bacteria. The astrocytes used in this study were derived from diverse sources, including induced pluripotent stem cell (iPSC)-derived astrocytes as well as primary and fetal astrocytes, as detailed in Table 1.

**TABLE 1:** Summary of RNA-seq datasets included in the meta-analysis of inflammatory responses in human astrocytes.

Dataset ID	Treatment	Dose	Cell source	Treated (*n*)	Control (*n*)	Platform
GSE147870	TNF-α	30 ng/ml	Fetal human astrocytes	9	9	Illumina HiSeq 4000
GSE147870	Poly(I:C)	200 μg/ml	Fetal human astrocytes	4	4	Illumina HiSeq 4000
GSE157461	TNF-α + IL-1α	30 + 300 ng/ml	Primary human astrocytes	5	5*	Illumina NovaSeq 6000
GSE157461	TNF-α + IL-1α + C1q	30 + 300 + 4000 ng/ml	Primary human astrocytes	5	5*	Illumina NovaSeq 6000
EMTAB11468	TNF-α	10 ng/ml	hiPSC-derived astrocytes	3	3	Illumina NovaSeq 6000
GSE160092	IL-1β	10 ng/ml	Fetal human astrocytes	2	2	Illumina HiSeq 4000
GSE166500	Palmitic Acid	200 μM	Primary human astrocytes	6	6	Illumina HiSeq 2000
GSE126750	TNF-α + IL-1α	10 + 3 ng/ml	Primary fetal astrocytes	3	3*	Illumina HiSeq 2500
GSE126750	IL-1β	3 ng/ml	Primary fetal astrocytes	3	3*	Illumina HiSeq 2500
GSE97619	IL-1β	3 ng/ml	Primary human astrocytes	2	2	Illumina HiSeq 2500
GSE97619	IL-1β	3 ng/ml	hiPSC-derived astrocytes	6	6	Illumina HiSeq 2500
GSE198722	SARS-CoV-2 (MOI = 1)	MOI 1	Primary human astrocytes	6	6*	Illumina HiSeq 2000
GSE198722	SARS-CoV-2 (MOI = 5)	MOI 5	Primary human astrocytes	6	6*	Illumina HiSeq 2000
GSE85143	Borrelia burgdorferi	MOI 10:1 (24 h)	Primary human astrocytes	3	3*	Illumina HiSeq 2000
GSE85143	Borrelia burgdorferi	MOI 10:1 (48 h)	Primary human astrocytes	3	3*	Illumina HiSeq 2000
GSE157509	IL-1β	10 ng/ml	hiPSC-derived astrocytes	7	7	Illumina HiSeq 2500
GSE120411	TNF-α	10 ng/ml	hiPSC-derived astrocytes	3	3*	Illumina HiSeq 2500
GSE120411	IL-1β	10 ng/ml	hiPSC-derived astrocytes	3	3*	Illumina HiSeq 2500
GSE120411	TNF-α + IL-1β	10 + 10 ng/ml	hiPSC-derived astrocytes	3	3*	Illumina HiSeq 2500
GSE120411	IL-6	100 ng/ml	hiPSC-derived astrocytes	3	3*	Illumina HiSeq 2500
GSE120411	FBS	2%	hiPSC-derived astrocytes	3	3*	Illumina HiSeq 2500
GSE120411	FBS	2%	Fetal human astrocytes	3	3	Illumina HiSeq 2500

Datasets retrieved from the Gene Expression Omnibus (GEO) and ArrayExpress/EBI repositories are summarized, including dataset accession numbers, inflammatory stimuli, treatment doses, numbers of treated and control samples, and sequencing platforms. Each row represents an individual treatment-control comparison. For datasets containing multiple experimental conditions, treatment groups are reported separately. Control sample sizes marked with an asterisk (*) indicate that the same control cohort was shared across multiple treatment comparisons within the corresponding dataset. TNF-α, tumor necrosis factor alpha; Poly(I:C), polyinosinic-polycytidylic acid; IL-1α, interleukin-1 alpha; IL-1β, interleukin-1 beta; MOI, multiplicity of infection; FBS, fetal bovine serum; hiPSC, human induced pluripotent stem cell.

### Read processing and mapping

After data cleaning and preprocessing, we observed that most samples were sequenced using Illumina HiSeq or Illumina NovaSeq platforms. Of the eleven datasets analyzed, seven employed a single-end sequencing strategy, while four used paired-end sequencing. On average, 32.7 million raw reads were obtained per sample, with an average GC content of 49% and read lengths ranging from 50 to 150 bp. After quality filtering (Q > 30), approximately 91% of the reads survived trimming with Trimmomatic. On average, only 1.87% of reads exceeded the *E*-value threshold in SortMeRNA, indicating a low level of ribosomal contamination, while 98.13% were classified as non-ribosomal. Mapping with STAR showed that, on average, 83.68% of the reads aligned uniquely to the reference genome, 9.18% mapped to multiple loci, 0.75% mapped to too many loci, and 6.4% remained unmapped (1.22% due to short length and 5.18% remained unmapped due to insufficient similarity to the reference or high biological noise. In total, 93.6% of the reads were successfully mapped. Gene quantification using HTSeq-count yielded an average of 6.17 million reads assigned to annotated genes per sample. These results (Supplementary Table 3) indicate that the RNA-seq data were of high quality, with low ribosomal content, good mapping performance, and reliable gene expression quantification across datasets. Gene-level counts were analyzed using limma-voom for differential expression within each dataset.

### Robust rank aggregation analysis

The RRA analysis was performed separately for up- and down-regulated genes using 22 ranked gene lists per regulation direction, generated from individual treatment-versus-control contrasts (Supplementary Table 4). From RRA were identified a total of 130 DEGs, of which 125 were upregulated and 5 were downregulated (Supplementary Table 5). The top 10 significantly upregulated genes were *SOD2, CXCL8, WAKMAR2, ELF3, VCAM1, PARP9, BIRC3, NFKBIA*, and novel lncRNA (ENSG00000288528), and the 5 downregulated genes were *IL17D, GDF10, REEP1, PSMB7* and *SKP2.* The expression patterns of these genes were visualized using heatmaps generated in Morpheus, which were stratified according to astrocyte source, including human induced pluripotent stem cell-derived astrocytes, fetal and primary astrocytes (Supplementary Figure 1).

### Functional enrichment and pathway analysis

**FIGURE 2:**
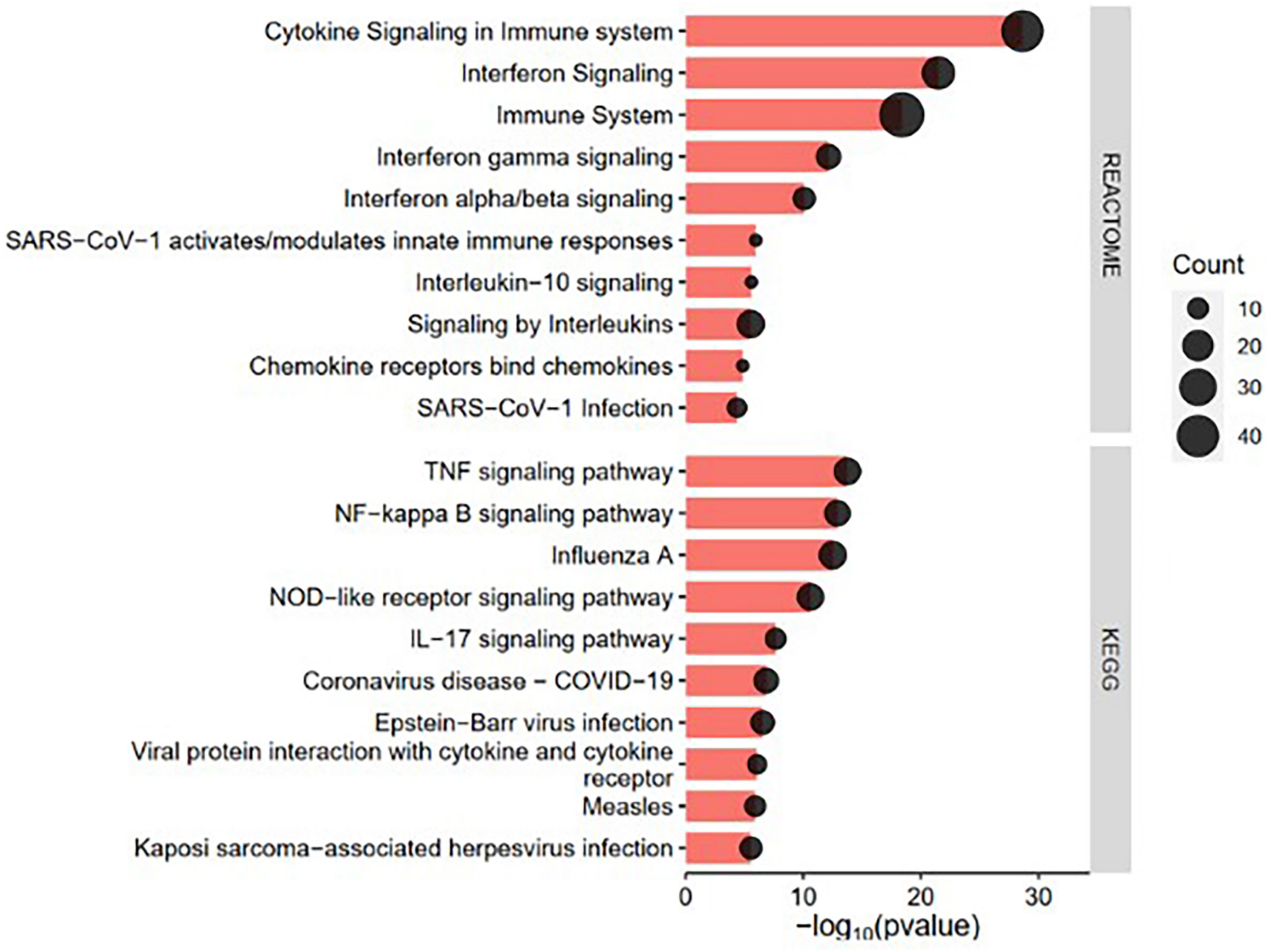
Functional enrichment analysis of the 125 consistently upregulated genes identified through Robust Rank Aggregation (RRA). Enrichment analysis was performed using g:Profiler across KEGG and Reactome databases. As no custom background gene set was specified; the default *Homo sapiens* background provided by g:Profiler was used. Significantly enriched pathways were identified after applying the g:SCS multiple testing correction at adjusted *p* < 0.05. Bar length represents enrichment significance expressed as -log10 (adjusted *p*-value), while dot size indicates the number of genes associated with each pathway. Pathways are grouped according to their source database (KEGG or Reactome). The enrichment profile highlights the overrepresentation of immune- and inflammation-related biological processes, including cytokine signaling, interferon signaling, TNF signaling, NF-κB signaling, and NOD-like receptor signaling.

The five downregulated genes identified (*IL17D, GDF10, REEP1, PSMB7*, and *SKP2*) were assessed for functional relevance; however, they did not show significant clustering within biological pathways related to inflammatory processes. In contrast, functional enrichment analysis of the 125 upregulated genes revealed significant activation of immune-related pathways, with prominent examples including “cytokine signaling in the immune system” and “interferon alpha/beta signaling” pathways. Additionally, pathways linked to viral infections, including SARS-CoV-1, influenza A, and COVID-19, were identified, indicating potential modulation of immune responses in these contexts. Other relevant pathways, including interleukin signaling, TNF, NF-kB, and chemokine receptor interactions, were also implicated, underscoring the critical role of immune pathway regulation in response to inflammatory stimuli (Figure 2).

**FIGURE 3:**
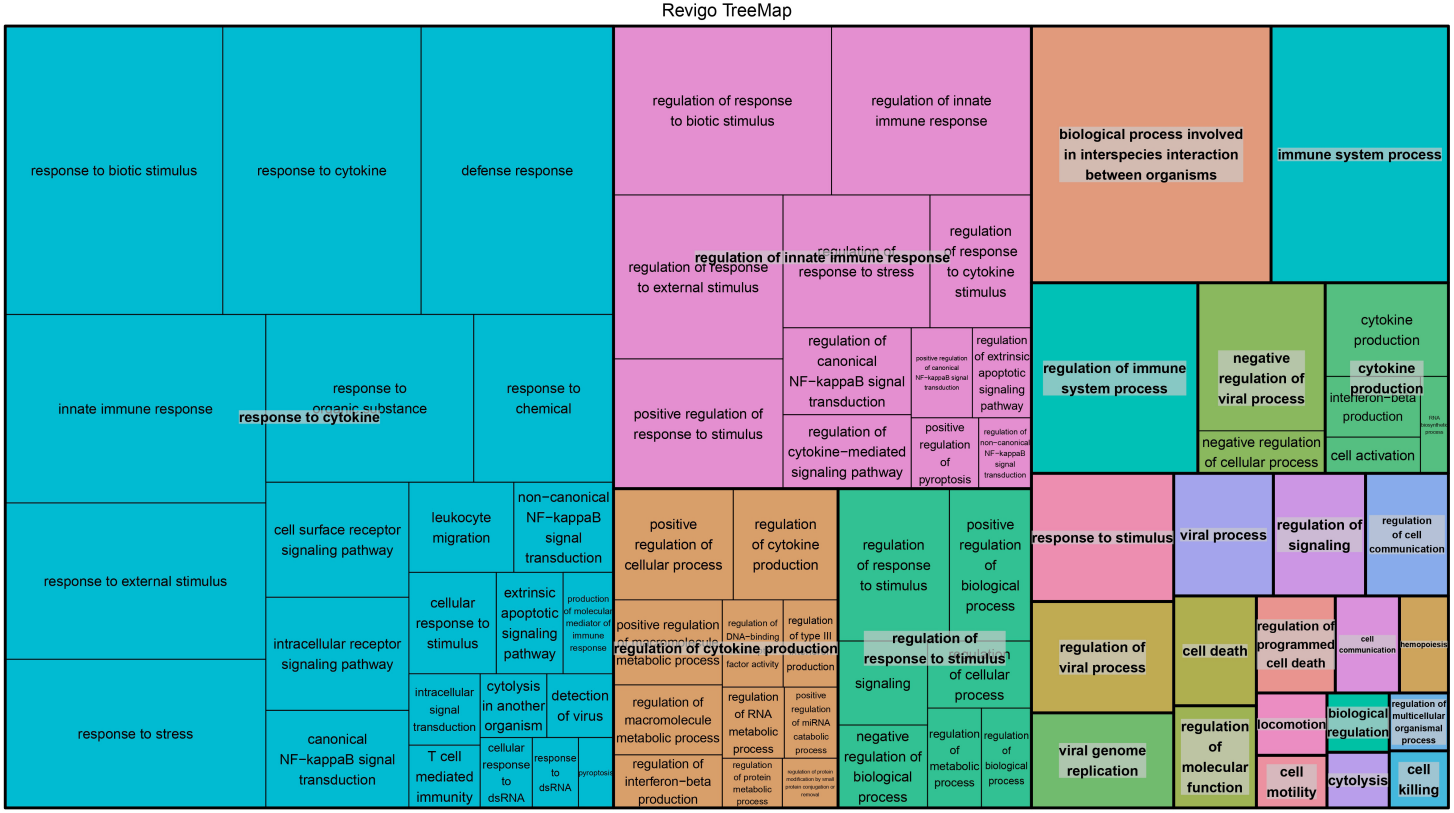
Treemap representation generated using REVIGO summarizing significantly enriched GO-BP terms obtained via the g: Profiler server. Each rectangle is a single cluster representative. The representatives are joined into “superclusters” of loosely related terms, visualized with different colors. Size of the rectangles corresponds to the *p*-value of the GO term.

Following the identification of 241 enriched GO-BP terms underlying the astrocytic response, we employed the REVIGO tool to resolve high semantic redundancy and identify representative functional clusters. By applying semantic similarity clustering algorithms, the list was condensed to 26 key biological processes (Figure 3). The 26 GO-BP terms encompass a diverse range of processes, including the regulation of immune system processes, cytokine production, response to stimuli, viral processes, and cell death. This analysis identified two main clusters: immune response and inflammatory response.

The biological processes associated with each cluster and their corresponding genes were then analyzed. Subsequently, genes shared between both clusters were identified, and a score was calculated based on the frequency with which each gene was associated with the different processes. Based on these scores, only genes with a relevant representation in one or both clusters were selected. This approach reduced the initial gene set to 33 genes, which were subsequently used for the construction of the protein–protein interaction (PPI) network.

### Protein–protein interaction network

**FIGURE 4:**
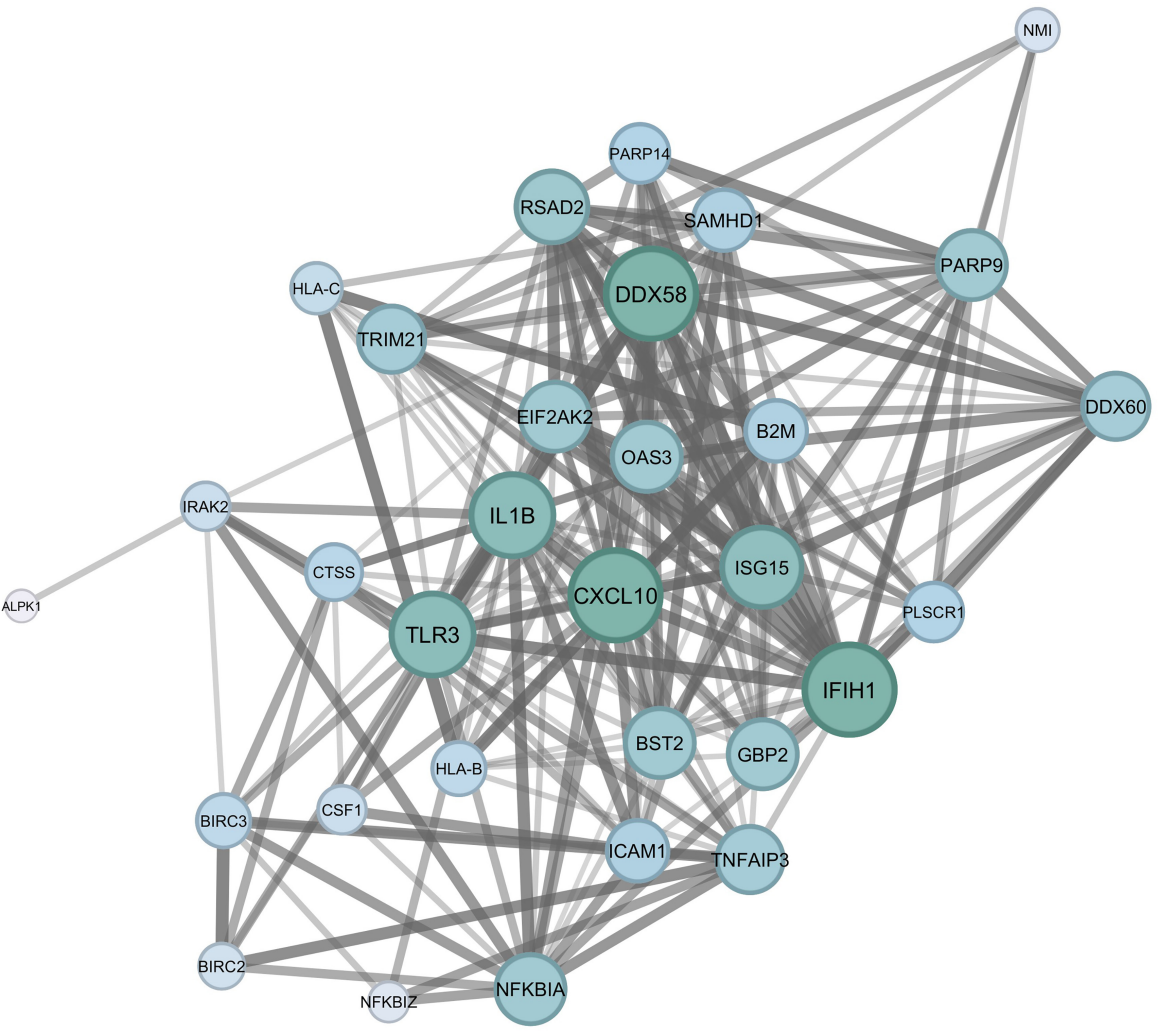
Protein-protein interaction (PPI) network of differentially expressed proteins involved in the regulation of inflammatory processes and immune system function. The network was constructed using the STRING database and visualized with Cytoscape software. Node size is proportionally mapped to the degree of connectivity. Edge opacity and thickness are scaled according to the STRING confidence score to prioritize high-confidence interactions. It comprises 200 edges, with a cluster coefficient of 0.711. The PPI enrichment *p*-value (<1.0e-16).

Because GO enrichment and REVIGO analyses consistently highlighted immune- and inflammation-related biological processes, we performed a targeted downstream analysis to identify a candidate immune-inflammatory core. Specifically, the 130 DEGs were filtered for genes annotated to immune and inflammatory response processes, resulting in a subnetwork of 33 nodes. These genes were then used to construct and visualize the PPI network via the STRING database and Cytoscape software. The resulting network (Figure 4) comprised 33 nodes and 200 interaction edges with an average node degree of 12.1, a cluster coefficient of 0.711, a network diameter of 3, and a *p*-value enrichment of less than 1.0e-16. To ensure the mathematical robustness of the inflammatory core, key nodes were defined using a multi-parametric topological filter. Nodes were prioritized if they ranked within top 15% of the distribution for both degree and betweenness centrality metrics. Based on these criteria, five primary nodes were identified: CXCL10, DDX58, IFIH1, IL-1B, and TLR3. These nodes exhibited the highest connectivity and centrality, suggesting their role as candidate hub nodes within the astrocyte-mediated inflammatory response. Comprehensive network parameters for each node, including eccentricity, average shortest path length, and topological coefficients, are detailed in Supplementary Table 6.

**FIGURE 5:**
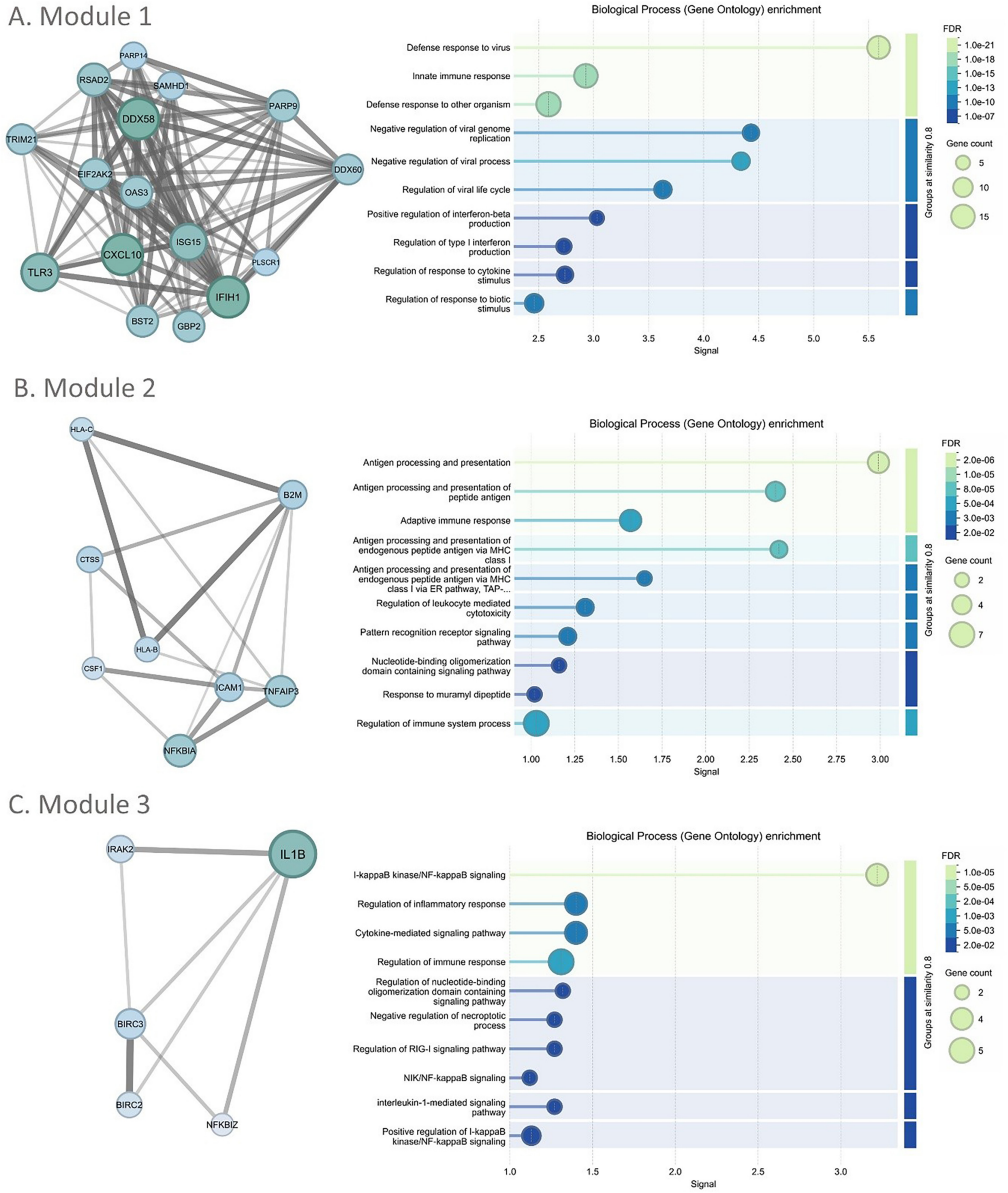
Functional module identification using MCODE. **(A–C)** Right panels show the corresponding dot plots for Gene Ontology (GO) Biological Process enrichment performed via the STRING database. Enriched terms were grouped based on a similarity threshold of ≥0.8.

Additionally, we used the MCODE plug-in to investigate whether associated proteins might form a highly connected molecular module. The MCODE clustering analysis identified three connected modules, ranging from 16 to 5 nodes. Module 1 was the most interconnected cluster (MCODE score = 13.87) with 104 edges and 16 nodes, identifying TLR3 as the seed node (Figure 5A). Enrichment analysis performed using STRING database revealed that the biological processes of this cluster are primarily associated with the activation of antiviral innate immune responses. Module 2 (MCODE score = 4.57) consisted of 16 edges and 8 nodes, with B2M as seed protein (Figure 5B). Enrichment analysis demonstrated biological process primarily involved in antigen processing and presentation via MHC class I, suggesting an activation of adaptive immune responses. Finally, the Module 3 (MCODE score = 3.5) showed fewer nodes and consisted of 5 nodes and 7 edges, with BIRC2 as seed (Figure 5C). Based on the enrichment analysis, the biological process identified focused on the activation of cytokine signaling and NF-κB pathway.

## Discussion

Astrocytes, the most abundant glial cells in the CNS, play a critical role in maintaining physiological homeostasis and responding to injuries or infections. While they remain in a homeostatic state under normal conditions, sensing pathological cues triggers a ‘reactive transformation’ characterized by finely tuned molecular and functional shifts (Kim et al., 2026). In inflammatory settings, these immunocompetent cells are essential for promoting both innate and adaptive immune responses. Specifically, they induce the expression of major histocompatibility complex class II (MHC-II) molecules and costimulatory signals, aiding peripheral immune cell recruitment (Le Thuc and García-Cáceres, 2024; Xie and Yang, 2015). Various inflammatory stimuli have been studied to clarify how astrocytes regulate these immune functions. For example, stimulation with LPS and Poly(I:C) triggers a significantly stronger innate immune response and effector molecule production than cytokine stimulation alone. Furthermore, pattern recognition receptor (PRR) activation by distinct viral or bacterial ligands modulates specific chemokine profiles according to pathogen type, underscoring how astrocytes tailor their responses to specific stimulus (McKimmie and Graham, 2010).

By employing Robust Rank Aggregation (RRA), we identified a candidate recurrent immune and inflammatory signature of 130 genes across 11 highly heterogeneous datasets. Within this signature, 125 genes were upregulated in response to a broad range of biological and biochemical proinflammatory stimuli (Supplementary Table 5). A large portion of these genes are associated with immune responses: 47 are specifically involved in immune regulation and 20 are linked to the induction of interferon alpha, beta, or gamma. These results align with previous studies that emphasized the role of astrocytes in the innate immune response, where they exhibit neuroprotective functions. To explore the biological significance of these 125 DEGs, we performed KEGG and REACTOME pathway enrichment analyses. These analyses revealed enrichment in key inflammatory pathways, including TNF (14 genes), NF-κB (15 genes), and NOD-like receptor (15 genes) signaling.

Previous studies have described enrichment in pathways related to immune system activation, antiviral innate immunity, major histocompatibility complex, cytokine/interferon signaling, and the inflammatory response through TNF signaling, NOD-like receptor, and NF-κB signaling in astrocytes exposed to similar conditions (Fields et al., 2022; Kim et al., 2022; Kong et al., 2022; LaRocca et al., 2021; Li et al., 2021; Rojas-Cruz et al., 2023; Santos et al., 2017; Vadodaria et al., 2021). Consistent with these observations, these pathways were also significantly enriched among the differentially expressed genes identified in our analysis. However, it is important to note that pathway enrichment analyses derived from transcriptomic data reflect coordinated changes in gene expression rather than direct measurements of protein abundance or signaling activity. Therefore, these results should be interpreted as indicators of transcriptional regulation of biological processes rather than definitive evidence of pathway activation.

TNF-α is a principal cytokine mediator regulating innate and adaptive immunity. Studies suggest that TNF-α can drive astrogliosis and play an integral role in the pathogenesis of various neurological disorders (Gao et al., 2024; Kim et al., 2022). NOD-like receptors (NLRs) are a specialized group of intracellular proteins that play pivotal roles in sensing molecules associated with intracellular infection and stress triggering signaling cascades that promote pathogen clearance. These receptors serve as scaffolding proteins that ease the assembly of diverse signaling complexes and modulate the activation of inflammatory caspases (Jorgačevski and Potokar, 2023). For instance, NOD1 and NOD2 in primary murine astrocytes recognize motifs of bacterial peptidoglycan and mediate IL-6 secretion in response to specific ligands (Giovannoni and Quintana, 2020; Sterka et al., 2006).

Notably, the NF-κB pathway was enriched with 15 genes, including *VCAM1, TNFAIP3, BIRC3, NFKBIA, CXCL2, ICAM1, CXCL5, CXCL1*, IL-1β*, CXCL10, CSF1, NFKB1, BIRC2, CXCL3*, and *TRAF1*. In the CNS, this pathway plays diverse roles depending on the physiological context, serving as a key regulator of the immune response, cell proliferation, and apoptosis (Wang et al., 2022). The NF-κB complex consists of five subunits that assemble in various combinations, and its activity is modulated by post-translational modifications such as O-GlcNAcylation and phosphorylation (Dong et al., 2023; Ridder and Schwaninger, 2009). NF-κB translocation affects cells differently depending on the location and duration of the signaling event. By promoting either pro-survival or pro-apoptotic states, it can ultimately exert neuroprotective or neurotoxic effects (Pozniak et al., 2014).

Functionally, NF-κB-regulated genes are primarily involved in immunoregulatory, inflammatory, and proliferative processes. Among the enriched genes, we found *TNFAIP3* which encodes a deubiquitinating enzyme that negatively regulates the TNF-induced NF-κB proinflammatory signaling pathway (Catrysse et al., 2014). Interestingly, NF-κB not only drives the expression of proinflammatory genes but also induces the transcription of its own inhibitors, such as TNFAIP3. This protein, deubiquitinates RIP1 and IKKγ, and inhibits the NF-κB-induced responses. This process forms a negative feedback loop that limits excessive inflammatory responses (Ruland, 2011).

Specifically, in astrocytes, NF-κB plays crucial roles in synaptic glutamate clearance, metabolic regulation and structural plasticity (Li et al., 2024; Pozniak et al., 2014). For example, NF-κB activation by TNF-α upregulates the production of nerve growth factor (NGF) and glia-derived neurotrophic factor (GDNF), highlighting its neuroprotective potential (Pozniak et al., 2014). However, under pathological conditions, chronic NF-κB signaling is associated with the transcription the transcription of downstream target genes, including TNF-α, IL-1β, IL-6 and iNOS, which promotes the expression and release of pro-inflammatory factors (Xu et al., 2021). Prolonged NF-κB activation can impair neuronal function and alter dendritic arborization morphology, contributing to adverse neurological outcomes (Kaltschmidt et al., 2005; Pozniak et al., 2014). In other mammalian species such as rodents, a chronic activation of astrocytic NF-κB promotes a loss of mitochondrial-associated proteins and the rise of inflammatory-related proteins (Jong Huat et al., 2024).

Previous studies have consistently identified NF-κB as a major regulator of the pro-inflammatory phenotype adopted by reactive astrocytes (Hasel et al., 2021; Jong Huat et al., 2024; Leng et al., 2022). In addition to its canonical function as a transcription factor, NF-κB act as a critical signal transduction mediator, linking extracellular inflammatory cues to the activation of downstream gene expression programs that shape astrocyte responses (Kaltschmidt et al., 2005; Pozniak et al., 2014).

Based on the 33 nodes of interest related to immune response and inflammation, a PPI network was constructed. The top five hub nodes with the highest degree of connectivity were CXCL10, DDX58, IFIH1, IL-1β, and TLR3. A remarkable finding of this meta-analysis is the consistent upregulation of the cytosolic sensors DDX58 (RIG-I) and IFIH1 (MDA5) across diverse non-viral inflammatory contexts, including stress induced by palmitic acid and systemic pro-inflammatory cytokine exposure. This could suggest that astrocytes adopt a phenotypic state of antiviral mimicry, engaging in ‘sterile’ inflammatory responses. Mechanistically, this state is likely orchestrated by the sensing of endogenous retroelements or mitochondrial DNA/RNA release under stress (Dhir et al., 2018; Russ and Iordanskiy, 2023), as seen in other neurological contexts (Blank and Prinz, 2017) Specifically, palmitic acid-induced mitochondrial impairment triggers metabolic and immune dysfunction, ultimately leading to antiviral mimicry. (Rojas-Cruz et al., 2023).

Both DDX58 and IFIH1, members of the RIG-I-like receptor family, play critical roles in the immune response by mediating the transcriptional induction of type I interferons. Although expression of these interferons is low under homeostatic conditions, it is significantly upregulated upon innate immune activation. Specifically, DDX58 acts as a double-stranded RNA receptor that, upon activation, triggers innate immune responses through mitochondrial antiviral signaling and the induction of interferon synthesis (Rehwinkel and Gack, 2020; Yoneyama et al., 2004). This interferon pathway, which was enriched in our analysis, is critical for promoting cell death in infected cells, activating adaptive immunity, and supporting hematopoietic stem cell renewal and proliferation (Wilkins and Gale, 2010). In neurotropic viral models generated from human astrocytes, DDX58 expression is constitutive and increases following infection, highlighting its role as an intracellular viral sensor (Furr and Marriott, 2012; Potokar et al., 2023). Additionally, the activation of RIG-I signaling in brain regions of patients with mild cognitive impairment may be involved in the accumulation of amyloid precursor proteins and in the exacerbated production of cytokines. This supports the hypothesis that this receptor is involved in the early events related to progression to Alzheimer’s disease (de Rivero Vaccari et al., 2014). In nonneuronal cells, DDX58 activation enhances autophagy, aiding in the clearance of lipid bodies that contribute to inflammation and subsequent apoptosis (Frietze et al., 2022). Moreover, IFIH1, which encodes the pattern recognition receptor MDA5, plays a complementary role by activating the TLR3 pathway via NF-κB, generating a distinct interferon signature. This activity is consistent with studies showing that astrocytes respond to the synthetic double-stranded RNA Poly(I:C) (De Miranda et al., 2009).

Like IFIH1, DDX58 is involved in canonical PRR signaling pathways, which interact by activating Toll-like receptor (TLR) pathways. In astrocytes, the expression of functional Toll-like receptors has been well documented, and their activation leads to the upregulation of genes encoding inflammatory mediators (Krasowska-Zoladek et al., 2007). Therefore, these receptors are considered key regulators of the innate immune response and are implicated in the progression of neuropathologies such as Alzheimer’s disease, Parkinson’s disease and amyotrophic lateral sclerosis (Kinsella et al., 2018). Members of the TLR family activate two primary signaling pathways, both of which depend on myeloid differentiation primary response gene 88 (MyD88), with the notable exception of TLR3, which utilizes an alternative signaling mechanism.

TLR3 has been described as a receptor expressed by human astrocytes under basal culture conditions. In addition to being activated by double-stranded RNA (dsRNA), TLR3 can also recognize endogenous mRNAs (Jack et al., 2005). Once activated, TLR3, along with the recruitment of other molecules such as TRIF (interferon inducer), initiates a signaling cascade that leads to the production of IFN-β and the activation of MAP kinases and NF-κB. This cascade promotes inflammation-related pathways, including those involved in glioma tumor progression (Krasowska-Zoladek et al., 2007; Li et al., 2017). In addition, TLR3 activation has been found to be related to cell death progression, with sex-dependent differences, where female cells induce apoptosis and male cells induce necrotic apoptosis (Chavez-Valdez et al., 2019).

Although TLR3 is expressed in various cell types, with its activation eliciting distinct cellular responses in each context, its activation in cultured astrocytes leads to prolonged inflammation (Seo et al., 2015). This inflammation results in the upregulation of several genes encoding cytokines, such as IL-1β, IL-1α, IL-6, and TNF-α, as well as chemokines, such as CXCL10, growth factors, enzymes, other inflammatory mediators and TLR receptors (Borysiewicz et al., 2009; Chavez-Valdez et al., 2019; Jack et al., 2005; Krasowska-Zoladek et al., 2007).

Our meta-analysis identified CXCL10 as a top-ranked hub node with high connectivity. Previous studies suggest it acts as a primary gatekeeper for the CXCR3-mediated recruitment of Th1 and NK cells during the astrocyte-driven neuroinflammatory response (Elemam et al., 2022; McKimmie and Graham, 2010). The CXCL10-CXCR3 axis functions as a regulatory interface between chemotactic recruitment and cellular reprogramming, ensuring that infiltrated Th1 and NK cells undergo metabolic and transcriptional preconditioning to withstand the neuroinflammatory environment (Bantug et al., 2018; Bufi et al., 2025; Corrado and Pearce, 2022; Koch et al., 2009). Transcriptionally, CXCL10 is regulated by external stimuli, including IFN-γ, TNF-α, and LPS, through NF-κB-dependent pathways (Vazirinejad et al., 2014). Its expression has been observed in neurons, glial cells, and stromal cells, where it can exert either protective or detrimental effects, depending on the etiology and context of neuroinflammation (McKimmie and Michlmayr, 2014).

Specifically, CXCL10 secretion from astrocytes has been shown to mediate T-cell recruitment to the brain under conditions such as multiple sclerosis and CNS viral infections (Cheng and Chen, 2014; Phares et al., 2013; Sørensen et al., 2002). Furthermore, during neuroinflammation induced by SCI, CXCL10 may play a role in disrupting the integrity of the blood–brain barrier, thereby fostering an environment that promotes the accumulation of peripheral immune cells and the amplification of immune responses. In this context, the release of CXCL10 by astrocytes may adversely affect oligodendrocytes and inhibit neuronal regeneration by attracting inflammatory cells, although the underlying molecular mechanisms remain to be fully elucidated (Qiao et al., 2022).

The critical and nonredundant roles of CXCL10 in neuroinflammation are controversial and have been examined across various models. For example, studies involving viral encephalitis, such as those involving multiple virus infections, such as mouse hepatitis virus (MHV) and West Nile virus (WNV), suggest that CXCL10 plays a beneficial role by enhancing the clearance of infected cells. Conversely, CXCL10-induced inflammation has been shown to be detrimental in conditions such as malaria encephalitis and trypanosomiasis (Amin et al., 2009; Campanella et al., 2008; Christensen et al., 2009). While numerous studies have linked CXCL10 expression to viral responses in the CNS, CXCL10 binding to the CXCR3 receptor promotes Th1 responses, generating positive feedback loops and facilitating the recruitment of microglia. Additionally, other studies have indicated that increased CXCL10 expression acts as an inflammatory mediator during the progression of neurodegenerative diseases and is correlated with the activation of ERK1/2 signaling pathways that mediate glial–neuronal interactions (Koper et al., 2018; Liu et al., 2014; Sui et al., 2006). Indeed, the overexpression of CXCL10 has been associated with neurotoxic processes attributed to increased intracellular calcium influx into mitochondria, which promotes membrane permeabilization and the subsequent release of cytochrome c, ultimately initiating caspase activation and leading to apoptosis (Sui et al., 2006).

Finally, IL-1β is recognized as one of the primary proinflammatory cytokines and plays a pivotal role in both the immune system and the central nervous system (Allan et al., 2005). Its presence has been associated with the maintenance of neuroinflammation, which contributes to the pathogenesis of Parkinson’s disease (Koprich et al., 2008). Pharmacological studies have validated its role in the progression of neuroretinal inflammatory responses, which lead to neural tissue damage (Dabouz et al., 2020). In Alzheimer’s disease patients, IL-1β has been identified alongside IL-1α, indicating the presence of active and persistent inflammation at the tissue level (Italiani et al., 2018). Under physiological conditions, particularly during aging, increased expression levels of TNF-α, IL-1β, and IL-6 have been observed in rat astrocytes via immunofluorescence techniques, a phenomenon not observed in neurons or microglia. These findings suggest that the astrocyte-driven loss of tissue homeostasis, characterized by a cytokine storm in the aging brain, may promote the development of neurodegenerative diseases (Willis et al., 2020).

The genes and pathways identified in this analysis provide a basis for generating hypotheses regarding astrocyte-associated inflammatory programs under pathological conditions. Rather than established therapeutic targets, mediators such as IL-1β, CXCL10, and NF-κB-related genes represent recurrent components of transcriptional inflammatory signatures across heterogeneous datasets. Further experimental studies are required to determine their functional relevance and potential implications in astrocyte-mediated neuroinflammation.

Overall, the findings of this study highlight the central role of astrocytes as immunocompetent mediators in response to inflammatory stimuli. The differential expression of genes involved in pathways such as TNF, NF-κB, and NOD-like receptors might reflects an activation of the innate immune system, characterized by the upregulation of proinflammatory cytokines and chemokines such as IL-1β and CXCL10, as well as viral RNA sensors like DDX58 and IFIH1. These results reinforce the active role of astrocytes in detecting damage- or pathogen-associated signals and in modulating the neuroimmune environment, positioning them as key contributors to brain inflammatory responses.

Several important limitations should be considered when interpreting these findings. Although the robust rank aggregation approach facilitates the identification of genes consistently regulated across independent datasets, the resulting signature may still be influenced by the characteristics of the studies included in the meta-analysis. In our analysis, the datasets comprised different astrocyte sources: iPSC-derived, fetal, and primary cells. While primary astrocytes exhibited a polarized response across a wide range of stimuli, fetal and iPSC-derived astrocytes showed higher sample-to-sample and stimulus-specific heterogeneity in their expression profiles (Supplementary Figure 1). Consequently, these differences in astrocyte origin, developmental maturity, experimental conditions, and dataset composition may shape the observed transcriptional patterns. Additionally, the inclusion of multiple treatment-control contrasts from the same datasets introduces a potential bias, as certain studies or stimulus categories may be overrepresented in the final RRA signature. For instance, because several viral and pathogen related datasets were included, interferon and antiviral signaling programs likely exerted an influence on the transcriptional profile.

The candidate inflammatory signature reported here should be interpreted within the context of the analyzed datasets and may not fully capture the diversity of astrocyte responses across all biological conditions. In addition, it is important to note that the candidate hub nodes identified based on network connectivity should not be interpreted as drivers of astrocyte inflammatory responses and have not been independently experimentally validated.

## Data Availability

The original contributions presented in the study are included in the article/Supplementary material, further inquiries can be directed to the corresponding author.

## Footnotes

1 https://github.com/karemra/Meta-analysis-astrocytes.git.

2 https://software.broadinstitute.org/morpheus/.

3 http://biit.cs.ut.ee/gprofiler/.

4 http://revigo.irb.hr.
