# Supplementary material for: Integrated meta-analysis of human astrocytes transcriptomes reveals a candidate recurrent inflammatory signature in response to inflammatory and immune stimuli
Source: Front Cell Neurosci. 2026 Jul 29;20:1870142. doi: 10.3389/fncel.2026.1870142 (PMC13461556; doi:10.3389/fncel.2026.1870142)
Supplement: Supplementary file 1 [file Data_Sheet_1.docx]

A. **iPSC-derived astrocytes	B. Fetal astrocytes**

**C. Primary astrocytes**

**Supplementary Figure 1 Heatmaps of RRA-derived differentially expressed genes across astrocyte populations.** Heatmaps showing the expression patterns of genes identified by Robust Rank Aggregation (RRA) across independent transcriptomic datasets of stimulated human astrocytes. Expression values were obtained from **limma-voom normalized RNA-seq data** and visualized using **Morpheus** <https://software.broadinstitute.org/morpheus/>**.** Columns represent individual samples and rows represent RRA-selected differentially expressed genes. Samples are grouped according to experimental condition and study of origin, as indicated by the annotation bars above each heatmap. Gene expression values were row-scaled using Z-score transformation to facilitate comparison across samples. The color scale represents relative expression levels, with blue indicating lower expression and red indicating higher expression relative to the mean expression of each gene. Heatmaps are shown separately for **(A) induced pluripotent stem cell-derived astrocytes (iPSC-derived astrocytes), (B) fetal astrocytes, and (C) primary astrocytes.**
